# Creating connections: developing an online space for cross-regional mentorship and network building in the dementia research field

**DOI:** 10.12688/amrcopenres.13091.2

**Published:** 2023-03-23

**Authors:** Josie Fullerton, Conceicao Bettencourt, Michael Daniels, Fiona Mclean, Susan Simpson, Adam Smith, Nathan Woodling, Fiona Kerr

**Affiliations:** 1 School of Cardiovascular & Metabolic Health, University of Glasgow, Glasgow, G12 8TA, UK; 2 Department of Neurodegenerative Disease and Queen Square Brain Bank, UCL Queen Square Institute of Neurology, University Collage London, London, WC1N 1PJ, UK; 3 UK Dementia Research Institute, College of Medicine and Veterinary Medicine, University of Edinburgh, Edinburgh, EH16 4SB, UK; 4 Division of Systems Medicine, School of Medicine, Ninewells Hospital & Medical School, University of Dundee, Dundee, DD1 9SY, UK; 5 Institute of Neurology, University Collage London, London, WC1N 3BG, UK; 6 Department of Genetics, Evolution and Environment, Institute of Healthy Ageing, University Collage London, London, WC1E 6BT, UK; 7 School of Molecular Biosciences, University of Glasgow, Glasgow, G12 8QQ, UK; 8 Department of Life Sciences, School of Applied Sciences, Edinburgh Napier University, Edinburgh, EH11 4BN, UK

**Keywords:** mentoring, mentorship, early career researchers, dementia, Alzheimer’s disease

## Abstract

**Background:**

Effective development and retention of talented early-career researchers (ECRs) is essential to the continued success of biomedical science research fields. To this end, formal mentorship programmes (where researchers are paired with one or more mentors beyond their direct manager) have proven to be successful in providing support and expanding career development opportunities. However, many programmes are limited to pools of mentors and mentees within one institute or geographical area, highlighting that cross-regional connections may be a missed opportunity in many mentorship schemes.

**Methods:**

Here, we aimed to address this limitation through our pilot cross-regional mentorship scheme, creating reciprocal mentor-mentee pairings between two pre-established networks of Alzheimer’s Research UK (ARUK) Network-associated researchers. We carefully created 21 mentor-mentee pairings between the Scotland and University College London (UCL) networks in 2021, with surveys conducted to assess mentor/mentee satisfaction with the programme.

**Results:**

Participants reported very high satisfaction with the nature of the pairings and the mentors’ contribution to the career development of mentees; a majority also reported that the mentorship scheme increased their connections outside of their home network. Our assessment of this pilot programme is that it supports the utility of cross-regional mentorship schemes for ECR development. At the same time, we highlight the limitations of our programme and recommend areas for improvement in future programmes, including greater consideration of support for minoritized groups and the need for additional training for mentors.

**Conclusions:**

In conclusion, our pilot scheme generated successful and novel mentor-mentee pairings across pre-existing networks; both of which reported high satisfaction with pairings, ECR career and personal development, and the formation of new cross-network connections. This pilot may serve as a model for other networks of biomedical researchers, where existing networks within medical research charities can act as a scaffold to build new cross-regional career development opportunities for researchers.

## Introduction

Mentorship is a key component of development for early-career researchers (ECRs, broadly defined here as researchers ranging from new PhD students to group leaders in their first years of independent research). Large-scale studies of ECR-focused mentorship programmes report quantifiable benefits for mentees^1^, including greater satisfaction with time management and higher measures of self-efficacy^2^. Within specific research fields, studies of mentorship programmes have demonstrated additional benefits to mentees. For instance, mentees participating in the international Children’s Oncology Group (COG) programme reported beneficial outcomes to career development, manuscript and grant preparation, and participation as members of field-specific committees^3^. Within dementia research, exemplar programmes such as the Carolina Center on Alzheimer’s Disease and Minority Research (CCADMR) also demonstrate clear benefits of mentorship for mentee success in achieving career milestones such as promotion or grant funding^4^. Mentorship provides benefits to mentors as well, with surveys reporting professional development benefits for mentors including enhanced communication skills and the development of improved leadership roles^5^. As one specific example, mentors participating in the COG programme noted above reported benefits including fresh perspectives, co-authored publications, and keeping mentors challenged and up to date^6^.

Diversity within mentor-mentee teams can provide additional opportunities as well as challenges. For example, just as culturally diverse teams of research paper co-authors are associated with higher impact^7^, cross-cultural mentorship pairings present unique learning outcomes that extend from problem-solving orientation to management styles and the role of cross-generational wisdom^8^. In addition to cultural diversity, geographic diversity between mentor-mentee pairings is a relatively underexplored area of opportunity for mentorship programmes. Connecting mentors/mentees from different regions is one way to ensure a larger mentor and mentee ‘pool’ to increase the likelihood of beneficial matches in experience and mentorship goals. In addition, cross-institute mentorship has the potential benefits of encouraging cross-institute research collaborations and developing field-wide mentorship cultures across institutes. Geographically diverse collaborations can be a particularly positive outcome, as research papers authored by international teams are cited more highly than those by single-nation teams^9^, and even collaboration among different research institutes within a single country improves the impact of the resulting papers^10^. Successful examples of cross-institute mentorship scheme include the National Research Mentoring Network among biomedical scientists in the United States; this network has been a pioneer example of capitalising on diverse experiences to address equity and inclusion for ECRs^11^. The success of these programmes suggests that there may be similar benefits for cross-institute mentorship programmes in other countries.

Despite this clear importance of cross-institution mentorship and establishing collaborations outside one’s own institution, opportunities to seek mentorship outside of one’s home institution can be limited for ECRs. This experience gap presents a particular challenge for ECRs and highlights the need for effective development opportunities to grow cross-regional ECR networks. Even greater challenges were faced by ECRs attempting to develop independent non-local networks during the global COVID-19 pandemic. For much of 2020 and 2021 in-person conferences were cancelled, leaving little opportunity for networking for ECRs outside of their own institution and harming prospects for career development^12,13^. One potential opportunity to grow these networks lies in mentorship programmes, such as those currently and successfully delivered in the UK by the Academy of Medical Sciences (https://acmedsci.ac.uk/grants-and-schemes/mentoring-and-other-schemes/mentoring-programme), British Neuroscience Association (https://acmedsci.ac.uk/grants-and-schemes/mentoring-and-other-schemes/mentoring-programme), and the Royal Society (https://royalsociety.org/grants-schemes-awards/mentoring-scheme/).

Within specific fields such as dementia research, there is a need to build on these examples to develop field-specific cross-institutional mentorship opportunities. To this end, we designed and carried out a pilot mentorship programme between two geographically distinct sub-networks of dementia researchers in the UK. This built on professional connections already established between members of the Scotland and University College London (UCL) Centres of the Alzheimer’s Research UK (ARUK) Research Network. ARUK’s Research Network for dementia researchers currently comprises individual centres for Scotland, Wales, and Northern Ireland, with nine regional centres in England including a London network that was divided into individual university networks until 2022. Among their activities, these networks each have specific programmes for ECR development; however, until recently these activities, including mentorship programmes, happened largely *within* network centres rather than *among* them. With the long-term goal in mind of creating a national cross-network mentorship scheme, we chose to start with a pilot scheme between two centres, as small-scale pilot programmes are a particularly useful way to allow time for surveying and interviewing participants to enhance future large-scale programme design^14^.

## Methods

### Making virtual links: establishing the mentoring scheme

The ARUK UCL-Scotland Mentorship scheme was established in late 2020 and launched in March 2021, through a collaboration between the Scotland and UCL ARUK Network Centre ECR Committees. Overall, the scheme was driven by ECR need and aimed to provide a formal platform to promote career conversations across the ARUK Network research community, with a strong focus on dementia research, professional and personal development, and network enhancement. These areas were identified through consultation with the ECR committees in both networks, as well as criteria used by successful mentoring schemes, such as that of the Royal Society^15^. Individual needs were also addressed by giving mentees opportunity in the application form to request any additional points that would be important to them for matching. This 6-month pilot was launched in 2020–21, with an initial focus on post-doctoral researchers and final year PhD students.

Taking advantage of the virtual environment created by the COVID-19 pandemic, the scheme was able to provide appropriate pairing across Scotland and London, as well as an online networking event and continued guidance and support to ensure mentors and mentees benefited from their partnership despite the geographical distances involved. Here we report on the challenges and successes of establishing this scheme, benefits to mentees and mentors, and suggestions for further development, with the aim of supporting a variety of career stages and mentorship across other networks in future.

### Mentor recruitment

To ensure support for the scheme from both Scotland and UCL Networks, the approach was to first identify potential mentors. Mentors were recruited on a voluntary basis by application form, with the criteria that they needed to be a member of either the Scotland or UCL ARUK networks and working at Senior Post-Doc/Junior Fellow level or above. A total of 32 mentors were recruited from Senior Post-Doc/Junior Fellow to Professor levels ensuring that a range of mentorship across career stages could be supported. Mentors were also asked to indicate their research expertise, as well as the areas in which they could provide mentorship from *‘Career and Research Advice’*, *‘Establishing Independence’*, *‘Building Networks & Managing relationships’*, *‘Equality, Diversity & Inclusion’ and ‘Work/Life Balance’*, to ensure that a variety of mentorship and research areas could be supported.

### Mentee recruitment

Applications were then opened to mentees. To aid pairing, applicants were asked to indicate the areas of mentorship, as above, that they would like support with, as well as their area of research, how the scheme would help them to develop their career in dementia research, why this scheme was valuable to their career, how they had been affected by the COVID-19 pandemic and to specify any other mentorship needs. A total of 21 mentees applied from final year PhD student to Senior Post-doctoral level. Due to the relatively small number of individuals, we chose not to collect EDI data during this pilot scheme in order to protect privacy.

For most areas of mentorship, the number of mentors who could offer experience tended to exceed the number of mentees in the reciprocal network requesting support. However, there was a slightly higher number of requests for *‘Building Networks & Managing relationships’* and *‘Establishing Independence’* from Scotland mentees than could be provided by UCL mentors. One area that was less well-supported and which had fewer requests from mentees was for *‘Equality, Diversity&Inclusion*’ (*EDI*). This likely does not reflect a lack of need in this area, but rather may reflect that the scheme was not specifically tailored to meet particular areas of support within this category.

### Pairing strategy

The mentor-mentee matching process is a key step in any mentorship programme, with potential strategies including semi-random allocation, self-selection by either mentees or mentors, open forum meetings to create pairings, and profile-matching by a programme leader or panel^14^. Among the strategies involving mentee selection of their own mentors, personal connections have been reported as some of the most important factors for mentee satisfaction with their pairing^15^, making these strategies less attractive for our purposefully cross-region scheme. In our programme we instead selected a panel of 5 programme organisers, two from each network and an independent member of the organisation Dementia Researcher who has wide links across the dementia research community, to employ a profile-matching approach, with a primary goal of ensuring that pairings were made across networks. We then focused particularly on the stated mentee objectives and goals for the mentorship experience to guide our selection of appropriate mentors. Initial matching was conducted by one member of the panel for each pair, based on areas of support required by the mentee, from the key areas above, overlapping research interests and any other specific needs requested. The whole panel then met to discuss each pairing individually and to ensure that the best matches were identified across the cohort. For fulfilling specific requests, around personal or professional development, knowledge of the mentors was required and this is where it was advantageous for the panel to include a wide range of experts in the field with both personal and collaborative connections. All mentees who applied in the pilot were paired with a mentor, and in 95% of cases this was from their reciprocal network.

Due to the relatively small number of individuals, we chose not to collect EDI data during this pilot scheme in order to protect privacy, but anecdotally 28% of all mentees specifically requested support from a female mentor or advice on navigating family circumstances. In order to align this with additional research and career development needs, in one case we sought a further mentor through our shared contacts and one mentee was given a choice of being paired with a male mentor in the reciprocal network or a female mentor in their own network and chose the latter. There were also specific requests for mentorship on clinical academic careers from UCL, and although we managed to fulfil these requests, we had to source one mentor outside of the original list and our pool of clinical mentors would need to be increased to ensure support in this area in future.

Mentees were then asked if they were satisfied with the pairing before mentors were informed. In a small number of cases mentees requested a change of mentor as the match was not directly within their research area, and in all cases a new mentor was sourced from the original list. Hence, having a panel with knowledge of the mentors available in both networks was beneficial in ensuring mentees were matched with those who could support their specific needs.

### Pilot launch

The mentoring period then ran between April and October 2021. A welcome meeting was organised, virtually, to provide information on expectations for mentoring, an opportunity for mentees to introduce themselves and build peer networks, and ECR support and mentoring talks from an ARUK Research Fellow and the ARUK Director of Research.

### Surveys and Feedback

We gathered feedback from participants through two separate surveys, one at the start and one at the end of the official mentoring period. At each stage separate surveys were provided to mentors and mentees using Microsoft Forms. All data was gathered anonymously and no personal data was collected as part of these surveys. As a service evaluation, ethics was not required for this work. Data was exported to Excel and are available at Figshare.

## Results

### Widening networks

**Figure 1:**
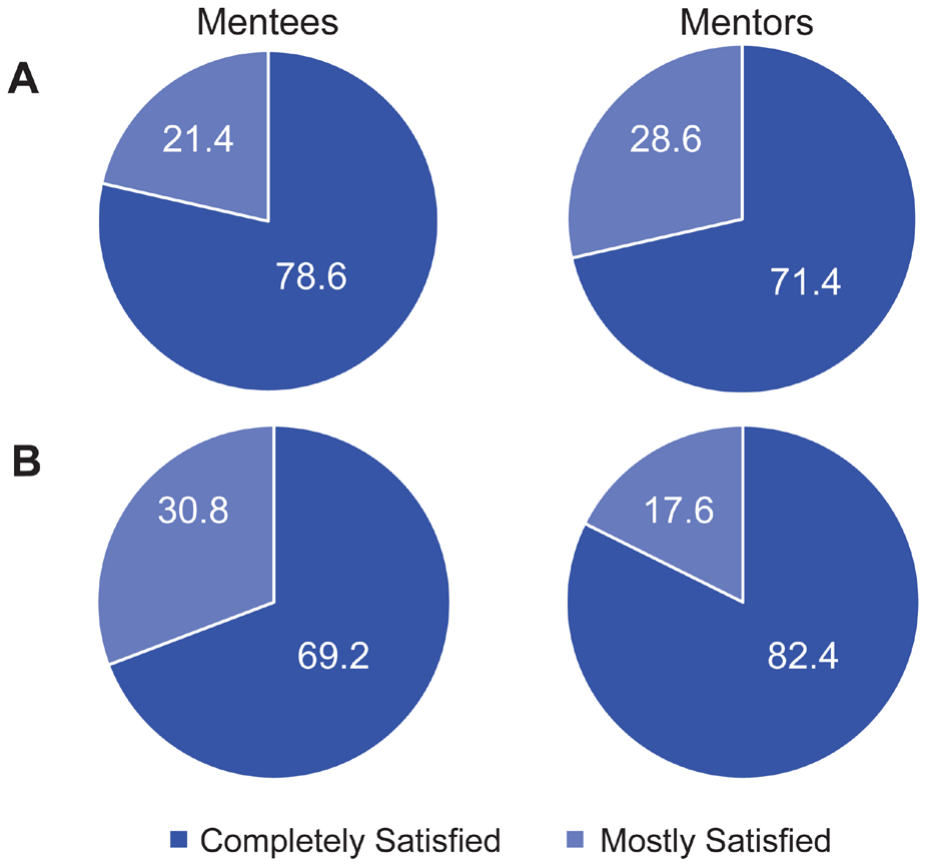
Satisfaction with the application and pairing process. Mentee and mentor responses to the question, “Overall are you satisfied with the application/pairing process of this scheme? (1- not satisfied, 2- slightly satisfied, 3- somewhat satisfied, 4- mostly satisfied, 5- completely satisfied)”, posed at the start (**A**) and at the end (**B**) of the mentoring period. Data are presented as a percentage of the total mentee or mentors scores for each category.

Reflections from participants For the initial survey 14 mentees and 14 mentors responded. Overall, mentees were satisfied with the application and pairing process (Figure 1A). Most appreciated having a variety of choices on the application to fulfil their mentoring needs, including key words for research and broad examples of areas of mentorship, and being consulted on the choice of mentor. Reciprocally, mentors were also satisfied with the recruitment/pairing process (Figure 1A), with some suggestions for inclusion of mentee biographies in future application forms.

At these early stages most had agreed some goals or areas that their mentee would like to work on, while others would have appreciated some guidance through the welcome/induction meeting and suggested this meeting should be held earlier in the process for future programmes.

Although most mentors and mentees were satisfied with the support and communication provided early in the mentoring period, there were requests for further networking and training events. During the welcome meeting we outlined expectations of and signposted to resources for mentors and mentees. Based on the feedback, however, training for mentors would be valuable in future programmes. To improve peer networking, we shared mentee introduction slides and contact details from the welcome meeting with participants via SharePoint.

**Figure 2:**
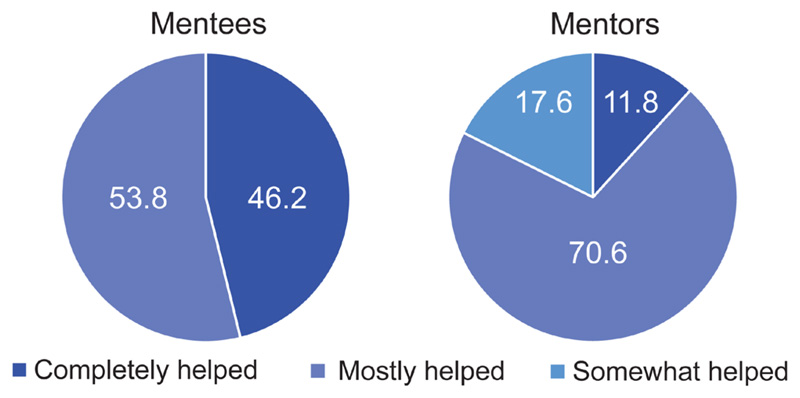
Support of the scheme for individual mentoring needs. Mentee responses to the question, “Do you feel your mentor has been able to help you to develop, or suggest ways of developing, in the areas that you identified for mentorship; and mentor responses to the question, do you feel you’ve been able to help your mentee to develop, or suggest ways of developing, in the areas that you identified for mentorship? (1- not at all, 2- slightly helped, 3- somewhat helped, 4- mostly helped, 5- completely helped)”. All data are presented as a percentage of the total mentee or mentors scores for each category.

For the final survey at the end of the 6-month mentoring period, 13 mentees and 17 mentors responded. Mentees reported high levels of satisfaction with the mentor-mentee pairing of the scheme (Figure 1B). All reported that their mentor had actively engaged in their mentorship during this period, and that they had helped them to both identify areas for support and to develop in these areas (Figure 2), and most felt that the scheme had fulfilled or surpassed their expectations. All mentees stated that they would recommend this type of scheme to other dementia researchers.

Mentors were also highly satisfied with the mentor-mentee pairings (Figure 1B). Interestingly, the percentage of mentees who were completely satisfied with their pairing decreased over time, whereas an increase in satisfaction rates was observed for mentors between the initial and final surveys (Figure 1A and B). Again, all mentors reported that their mentee had been actively engaged in their mentorship and that they had identified areas for support and development. Contrary to mentees, fewer mentors felt that they had completely helped their mentee to develop (Figure 2), which may reflect the mentoring training required as identified above and again in the final survey. All mentors, and all mentees except one, reported that their mentoring relationship was still active at the end of the mentoring period. Encouragingly, 94.1% of mentors said they would volunteer as a mentor if the scheme ran again.

**Figure 3:**
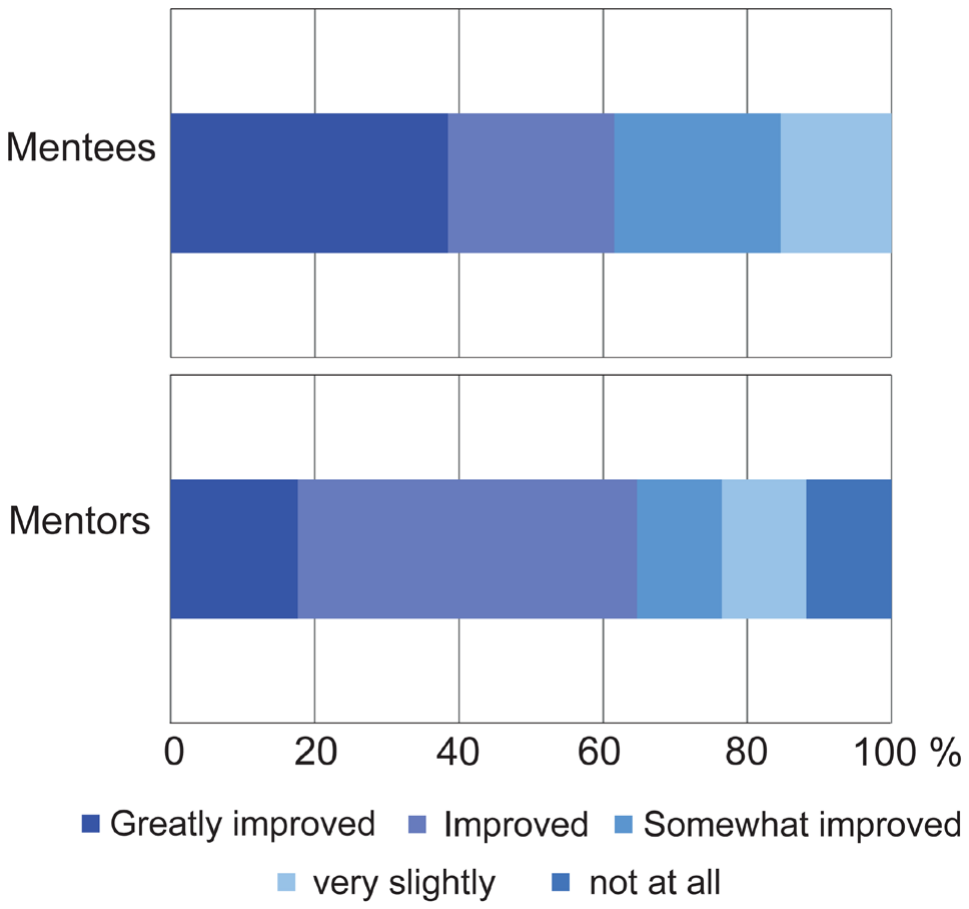
Cross-Networking success of the scheme. Mentee and Mentor responses to the question, “How well do you think this programme expanded your connections outside of your ‘home’ network? (1- not at all, 2- very slightly, 3- somewhat improved, 4- improved, 5- greatly improved)”. Data are presented as a percentage of the total mentee or mentors scores for each category.

One of the main aims of the scheme was to expand networks outside of local areas. For mentees, the majority (61.5%) felt that their connections outside of their home network had been greatly improved or improved (Figure 3). Interestingly, most mentors (64.6 %) also felt that the programme had expanded their connections as per improved or greatly improved responses (Figure 3). Hence overall, despite the geographical distance, the cross-network mentoring scheme did facilitate networking outside local areas for both mentors and mentees. Further improvements could be made, however, if at least one in-person event could be facilitated, and improved platforms for mentors and mentees to share information were implemented, as suggested by mentors in the final survey.

## Discussion

Through this pilot scheme, we aimed to co-ordinate mentee-mentor pairings for ECRs utilising two geographically distinct and established sub-networks of ARUK researchers, from the ARUK Scotland and UCL Research Network Centres. Specifically, this programme aimed to create new opportunities for cross-regional mentorship, enabling advice less likely to be biased by intra-network relationships, as well as highlighting potential collaborations for ECRs outside of their own institution, which can be difficult to establish organically.

Building upon pre-established ARUK networks, our pilot scheme consisted of 20 mentee-mentor cross-centre pairings, and one internal pairing. As previously discussed, we limited the number of pairings to allow us to carefully survey and interview participants, to enhance the implementation of our full-scale programme^14^. Although the definition of a successful mentorship match is difficult to define, our survey indicated that our pairing strategy was positively received, as all mentees and mentors stated that they were either ‘satisfied’ or ‘completely satisfied’ with their pairings. As indicated above, however, we observed a decrease in complete satisfaction with the pairing for mentees but an increase for mentors over time. It is unclear what might be driving this trend, which on reflection could have been addressed by including use of open text fields in relation to this question in our survey design. One possibility is that the value of the mentoring relationship to mentees decreased after the initial meeting for some pairings. This may be in line with Kathy Kram’s model of mentoring as a dynamic process^16^, whereby after the initiation and cultivation stages of building a mentoring relationship, the benefits to the mentee inevitably decline due to either a change in the structure of the relationship or because their initial support needs have been met. But this natural progression is more likely to take place over a longer period than that included in this pilot scheme. Alternatively, the slight reduction in mentee satisfaction may also reflect that some mentors were less experienced, and that further training needs to be supported in future.

Mentorship, separate from academic supervision or annual appraisals, is a vital element of ECR training, providing one-to-one support for newly qualified postgraduate researchers (PGRs) or for those undergoing professional development^17^. Again, we assessed our pilot scheme in relation to ECR career development and progression; all mentees reported that their mentor ‘completely helped’ or ‘mostly helped’ with their career development and personal goals. Similarly, many mentors (82%) reported that they ‘completely helped’ or ‘mostly helped’ their mentees, although there was a small number who felt that they hadn’t helped their mentee at all. Again, limitations in our methods by not including open text responses in relation to this question make it difficult to ascertain the reason for this. It may be reflective of the training needs discussed above, or that the style of mentorship adopted leaned more towards a one-directional coaching approach, therefore leaving less room for discussion and feedback from mentees^18^. Current literature demonstrates that successful mentorship is associated with increased career satisfaction and productivity^18,19^; which further highlights the importance of careful programme planning and appropriate mentorship pairings.

As geographic diversity of mentor-mentee pairings is a relatively underexplored area of opportunity for mentorship programmes, we aimed to highlight the need for cross-regional ECR networks and assess improved connections beyond ECRs own institutions. Importantly, our pilot programme demonstrated that the majority of mentees and mentors ‘greatly improved’ or ‘improved’ connections outside their institute. We harnessed the virtual and remote working nature created by the COVID-19 pandemic, which in turn increased the ‘pool’ of mentors, broadening the range of experience, diversity, and creating greater opportunities for ECRs – a benefit that can be seen in the high satisfaction scores for the pairings reported by both mentors and mentees. An additional potential benefit of cross-centre mentorship pairings is that they can greatly reduce bias, allowing ECRs to speak freely without prior judgment^20^; although our pilot surveys did not include a metric of this outcome.

This pilot illustrated that both mentees and mentors gained positive interactions from the scheme, and importantly the programme is well-supported by the ongoing commitment of mentors across a variety of research disciplines, who have experience in supporting a broad range of areas for mentorship. This is in line with other mentorship schemes where mentees reported quantifiable benefits, including greater satisfaction with time management and higher measures of self-efficacy due to their pairings^2^.

## Pilot limitations

Although our pilot demonstrated a range of success, we believe this scheme has areas to improve prior to a full-scale roll out of a cross-network mentorship programme. One prominent limitation of our pilot programme was our small number of pairings and the inability to effectively account for areas of EDI; this area was less well-supported by mentors and had fewer requests from mentees. Although academic institutions have increased their recruitment of EDI champions across specific demographics; EDI support must also include methods and advice to challenge current structures and systems that allow injustice and inequality to thrive. This will involve uncomfortable conversations and rigorous monitoring of ongoing processes to fix and advise on deep-rooted problems, which is often difficult to discuss in an informal mentorship setting. Yet this is a particularly important shortcoming that should be considered for future mentorship schemes, as multiple studies of mentorship programmes describe self-reported unfulfilled needs for participants from minoritized backgrounds, as well as a lack of consideration for intersectional identities^21^. To this end, future large-scale mentorship schemes can draw on the recommendations from reviews of the mentorship literature^21^ and programmes such as the National Research Mentoring Network that have successfully implemented mentorship training schemes aimed at EDI^11^.

Regarding increasing connections, we did not further dissect the responses from mentees who reported that they did not improve their connections out of their institute. For future methodologies, it would be useful to determine why this was reported, and how this could be improved. It could be due to the focus of the discussions, which should be ascertained, or perhaps a further follow-up questionnaire would determine if a longer period of time allowed this to develop.

An additional limitation of our pilot scheme was the lack of formal mentorship training provided to mentors, which is essential to enhance guidance exchanged between mentor and mentee, but also assist mentors in their own development^22,23^. For this pilot scheme to be scaled up to include additional regions, we would recommend that both EDI and mentor training be considered in the planning of the programme.

## Future directions

Mentoring is a vital way to support ECRs and has been highlighted as a preferred method of receiving careers support^17^. Following the success of the current pilot mentorship scheme, ARUK agreed to support, fund, and administer the design of a full-scale ARUK mentorship scheme pairing mentors and mentees across all Research Network Centres. This would be open to all ARUK Network Members (Membership is free for Biomedical and Clinical Dementia Researchers across the UK). From our findings here, we highlighted areas of improvement to better develop the full mentorship scheme. Firstly, although our pool of mentors provided a range of experience from two of ARUK’s network centres, by recruiting from a wider range of sub-networks we aim to further support areas of EDI such as race, gender, and disability, and to provide mentorship for all levels of PhD students, Post-Doctoral Researchers and Clinical Academic careers.

Further, we believe that the skills or practices utilised by the mentors that positively influenced the mentees should be discussed and reported, as this would allow the scheme to further develop and aid the evolution of guidance material received by the pairing at the start of the programme. For detailed analysis, ethical approval should be collected prior to survey design, to allow reporting of data reflecting the reasons why a mentee or mentor may perceive the programme as ‘satisfying’, or why mentors perceived themselves as less helpful than mentees perceived them. It would also be useful to collect longitudinal data, to determine how many pairings remained in contact, if cross-centre connections developed and if mentors/mentees registered to the scheme again. Importantly, EDI monitoring data should be included to allow programme organisers to assess the demographical spread, but also the guidance received by those who requested EDI specific support.

Finally, we suggest that future programmes could benefit from more innovative approaches to mentorship, specifically by delivering mentorship pairings across career pathways within dementia research, such as collaboration with mentors from biotechnology, pharmaceutical sector, or other industries. It is also essential that mentors are provided specific mentorship training, as it would be useful to build confidence in new mentors and to ensure quality of mentorship across all mentor/mentee interactions. Although harnessing the online nature of the COVID-19 pandemic was useful, we hope to create further networking opportunities for mentees and mentors in-person or through more interactive online platforms such as Gather town (https://www.gather.town/), which would also greatly improve the success of the scheme to foster connections outside of ‘home’ networks not only with more senior mentors, but with peer-mentors as well.

## Conclusions

Our pilot mentorship scheme allowed us to generate novel mentee-mentor pairings across pre-existing ARUK Scotland and UCL networks. Both mentors and mentees in this pilot scheme reported high satisfaction with the nature of the pairings, the programme’s ability to help develop ECR career goals and personal development, and the formation of new cross-network connections. Although there are clear limitations of this small pilot scheme, we believe that with the improvements suggested above, the new full-scale mentorship scheme to be implemented by ARUK has the potential to contribute to the career development of dementia researchers in the following ways:

- provide career development and support for ECRs early on in their careers, particularly PhD students and clinical academics
- provide career development and support for ECRs in minoritized EDI areas
- connect individuals in smaller academic communities with more limited local support to a broader and more geographically diverse network or researchers
- develop new collaborations within and between academia and industry

Combined, this approach can further improve the support and development of early career dementia researchers. It may also serve as a model for other networks of biomedical researchers working on other disease-focused areas, where existing networks within medical research charities can act as a scaffold to build new cross-regional career development opportunities for researchers.

## Data Availability

All numeric data from the surveys associated with this programme are provided in their raw form in this article and information was collected anonymously. Data protection safeguarding was employed for mentor and mentee application forms. This was through consent, via privacy notice, for the collection of data for mentor-mentee pairing purposes only, and this data is therefore not available or reported as part of this article. No other data are associated with this article. Figshare. ARUK Pilot Mentoring Scheme Survey Data. DOI: https://doi.org/10.6084/m9.figshare.c.6112032.v1^24^ Data are available under the terms of the Creative Commons Zero “No rights reserved” data waiver (CC BY 4.0 Public domain dedication).

## *Revised* Amendments from Version 1

The authors greatly appreciate the comments the reviewers have made, which we believe will further strengthen this manuscript. Combined, the authors have made the following changes to the original manuscript. Introduction: the opening paragraph in the introduction has been edited to focus on the need of collaboration and supporting structures, to lead into learning, training and professional development of early career researchers. Methods: as directed by the reviewer, the authors edited the methods section to better describe the establishment of the scheme, how the mentors/mentees were recruited, and the pairing strategy of the mentees-mentors. Results: as discussed by the reviewer, the authors agree that figure 1 and 2a would be better displayed as a single figure; the manuscript and figure legends have been adjusted to reflect this. Discussion: the reviewer raised the point that a number of items mentioned in the results have not been adequately assessed in the discussion. Therefore, to the discussion we have added some text regarding the satisfaction of pairing, the limitations of our methods to dissect this information, and requirements for training based on this. Further, we added a section regarding the limitation of our pilot’s ability to support ‘equality, diversity and inclusion’ areas and how best to improve this. A further study limitation, which we added text about, is the schemes ability to increase connections. Due to the nature of our surveys, we did not dissect this information; however, future studies should determine this. The authors believe that these changes further strengthen the manuscript and are grateful for the time the reviewers spent reading and assessing our work.

**Any further responses from the reviewers can be found at the end of the article**

## Open Peer Review

**Current Peer Review Status:** ✔ ✔

**Version 2**

Reviewer Report 27 March 2023

https://doi.org/10.21956/amrcopenres.14395.r27105

**© 2023 Guccione K.** This is an open access peer review report distributed under the terms of the Creative Commons Attribution License which permits unrestricted use, distribution, and reproduction in any medium, provided the original work is properly cited

✔ **Kay Guccione** http://orcid.org/0000-0001-9910-9388

Head of Research Culture and Researcher Development, Research & Innovation, University of Glasgow, Glasgow, UK

Many thanks to the authors for their careful consideration of my feedback, and for working to produce this updated version. I am really happy to see this paper sharing insider insight into the developmental value of mentoring and discussing the challenges of programme leadership and evaluation. A useful addition to the literature.

No competing interests were disclosed.

***Reviewer Expertise: Researcher Education and Development, Mentoring, Service Evaluation***

**I confirm that I have read this submission and believe that I have an appropriate level of expertise to confirm that it is of an acceptable scientific standard.**

**Version 1**

Reviewer Report 18 November 2022

https://doi.org/10.21956/amrcopenres.14170.r27013

**© 2022 Guccione K.** This is an open access peer review report distributed under the terms of the Creative Commons Attribution License, which permits unrestricted use, distribution, and reproduction in any medium, provided the original work is properly cited.

** ?** **Kay Guccione** http://orcid.org/0000-0001-9910-9388

Head of Research Culture and Researcher Development, Research&Innovation, University of Glasgow, Glasgow, UK

Dear Authors,

Thank you all for submitting this work. Your paper focussed on the conceptualisation and piloting of a cross-regional mentorship programme offering insight for others who are seeking to initiate a similar programme. Coming to your paper from a career of designing and managing mentoring programmes for researchers, I think you have created an effective pilot programme, making good design decisions, and the limitations and unknowns you acknowledge are astute and valid.

My view is that this paper’s value to the scientific community lies in how it can inform others’ practices in designing and supporting a mentoring programme that operates across regions. In saying this, two things strike me as of particular use to the community: firstly, the discussion of your observations of the value the programme offered in terms of ECR development, and also (importantly) the subsequent limitations of the study (what is not known). And secondly, the links you are making between mentorship and collaboration. It is with highlighting these two contributions in mind that I have written the recommendations below.

There are of course many commendable and insightful parts to your paper but for brevity in this report, I’ve only mentioned the unclear points or offered ideas for you to consider and action to increase the impact of the paper. Most are minor, but some warrant your thoughts. I offer my comments in the spirit of providing a developmental review experience, rather than a list of demands, and I am happy to have a dialogue if any of my suggestions or questions are unclear.

**Larger recommended edits:**

Overall, your paper shows that/how a cross-regional mentoring programme can be built to develop early career researchers. Your design, data and discussion align with this. However, your opening paragraph in the Introduction sets up the idea that the mentorship programme will demonstrate an impact on research collaboration. I had expected on reading this to see data on how many of the mentoring pairs went on to collaborate on a research project, or some discussion of the potential for collaboration through the increased professional networks gained through mentoring. After leading with the need for collaboration, and structures that support collaboration, you move into concepts of learning, training, and professional development. I think this opening section would have a greater impact if the links between mentoring, professional development and collaboration were made explicitly. Or, as an alternative, if the section about collaboration was minimised in favour of including more literature on the benefits of mentoring for career development.

It would be of practical value to the readers if you make the distinction between ‘Pilot Limitations’ (programme limitations and recommended developments) and ‘Future Directions’ (research limitations, what is not known, i.e. recommendations for evaluation of mentoring). This would help you avoid repetition in these sections and would bring a new dimension of learning for the readers. I suggest that the ‘Future Directions’ section could usefully discuss the ideas of: what skills or practices were deployed by the mentors that created value for the mentees; the collection of additional data that helps to explain why a mentee or mentor may perceive the programme as ‘satisfying’ or beneficial, and why this might change over time; why mentors perceived themselves less useful than mentees perceived them; and, the inclusion of diversity monitoring data in the analysis, that would enable you to show ‘who does the work of mentoring’. I say a bit more about this in the comments below related to the findings.

**Smaller recommended edits:**

*Abstract*

This sentence is unclear: “Our assessment of this pilot programme is that it supports the utility of cross-regional mentorship schemes for ECR development.” – I think you mean e.g.: Our assessment of this pilot programme is that it supports the **application** of cross-regional mentorship schemes to **enable** ECR development.

Word substitution/addition suggestion: “In conclusion, our pilot scheme generated successful and novel mentor-mentee pairings across pre-existing networks; **both mentors and mentees** reported high satisfaction with pairings, **gains for** ECR career and personal development, and the formation of new cross-network connections.”

*Introduction*

Para 2, for example. Commonly the word ‘development’ would be used with mentoring (to indicate a bi-directional, non-didactic learning method) rather than ‘training’.

Unclear sentence:"Connecting with mentors/mentees from different regions is a potential starting point for cross-institute collaborations, while providing the additional benefit of a larger mentor and mentee ‘pool’ for greater diversity in experiences and mentorship goals." - I think you are saying: …while providing the additional benefit of a more diverse mentor and mentee ‘pool’ to increase the likelihood of a productive match. – or something similar?

When you say:"Among their activities, these networks each have specific programmes for ECR support; however…", I suggest replacing ’support’ with ’development’, to maintain the focus on the professional/career development aspects you later go on to name in the matching process.

*Methods*

Here: "Overall, the scheme was driven by ECR need and aimed to provide a formal platform to promote knowledge sharing across the ARUK…", I suggest saying explicitly how you consulted ECRs on their ‘needs’ even if the answer is a simple ‘through informal conversations’. Perhaps you also drew on the information on mentee application forms, or drew from e.g. the Researcher Concordat, or the Researcher Development Framework? Both these docs mention mentoring and it could be good to include them.

In the above, the phrase"promote knowledge sharing" implicitly implies a pedagogical model for mentoring based on knowledge or advice – was this intentional? If not, a phrase like to promote ‘career conversations’ would generalise the activities that come under ‘mentoring’.

Mentor recruitment: “mentors were asked to register” - were they volunteers or did someone (e.g. line manager) require specific individuals to register?

Mentee recruitment: The phrase “why this scheme was attractive” seems unfocused. I imagine the word ’attractive’ in this case is meaning ‘valuable to their career’. Perhaps it was worded like this on the form though?

This sentence is unclear: “Mentor support for most areas requested tended to exceed that requested by mentees in the reciprocal network, except for a slightly higher number of requests for *‘Building Networks&Managing relationships’* and *‘Establishing Independence’* from Scotland mentees than could be provided by UCL mentors.” Perhaps use the idea of what the mentors ‘offered’ rather than what they ‘requested’.

On EDI development requests, it could be good to come back to this in the discussion and note the relative difficulty of asking for this kind of help.

Suggest either removal of the sentence “This event was well-received, with open and honest discussion.” – or if it’s pertinent to what follows, elaborate on the content of the discussion, and what the ability to have an open discussion means for the effectiveness of mentoring, e.g. participants trust each other, and the scheme organisers.

*Results*

Suggest removal of “To improve peer networking, we shared mentee introduction slides and contact details from the welcome meeting with participants via SharePoint.” – or move it to the Methods.

This comment is interesting and should be picked up in the discussion in relation to Fig 2B: “Contrary to mentees, fewer mentors felt that they had completely helped their mentee to develop…” – one reason for this could be as you say, the ability to recognise good practice based on training. Another could be that they did not get validatory feedback from their mentees. Another is that they used a more ‘coaching’ style rather than advice, which, being less of a ‘transaction’ can often open up an idea for mentees that comes to fruition after the meeting. This links to my comment about not knowing about the nature of the skills/approaches used by mentors – a methodological gap for further study.

Fig 1 and Fig 2: I wanted to see the satisfaction start/end charts (Fig 1 and Fig 2A) presented as one figure (with Fig 2B separately, as in my point above) This would highlight something additionally interesting that you could then pick up in the discussion – the fact that for some mentees, satisfaction decreased over time. But for mentors, it increased. Why might this be? What underlies this? Some open text data may have supplemented this gap and should be acknowledged in the discussion. One idea for explaining this is that for some pairs, after the initial meeting, value decreased (see Kathy Kram’s model of mentoring that shows value decrease over time). Another explanation is that some of the mentors were less skilled, and this would support your argument for training.

Fig 3 and data: The figure is logical in that it shows mentors made less gain than mentees, which could usefully be acknowledged explicitly in the text. In the text, you say “For mentees, the majority (61.5%) felt that their connections outside of their home network had been greatly improved or improved (Figure 3). Interestingly, most mentors (64.6 %) also felt that the programme had expanded their connections as per improved or greatly improved responses (Figure 3).” – this leaves me with questions about why the remainder didn’t think it added connections. You don’t present any qualitative data that could help explain this, and since it’s a question your readers will have, this gap should be acknowledged as an area in which the evaluation methodology could be developed.

*Discussion*

The word ‘bias’ needs to be explained in this sentence. “An additional potential benefit of cross-centre mentorship pairings is that they can greatly reduce bias, …” In this context, bias towards what? Perhaps ‘conflict of interest’ describes what you mean?

[see points above related to the discussion of Results]

**Is the work clearly and accurately presented and does it cite the current literature?**

Partly

**Is the study design appropriate and is the work technically sound?**

Yes

**Are sufficient details of methods and analysis provided to allow replication by others?**

Yes

**If applicable, is the statistical analysis and its interpretation appropriate?**

Not applicable

**Are all the source data underlying the results available to ensure full reproducibility?**

Yes

**Are the conclusions drawn adequately supported by the results?**

Partly

**Reviewer Expertise:** Researcher Education and Development, Mentoring, Service Evaluation

**I confirm that I have read this submission and believe that I have an appropriate level of expertise to confirm that it is of an acceptable scientific standard, however I have significant reservations, as outlined above.**

Reviewer Report 01 November 2022

https://doi.org/10.21956/amrcopenres.14170.r27010

**© 2022 Baertschiger R.** This is an open access peer review report distributed under the terms of the Creative Commons Attribution License, which permits unrestricted use, distribution, and reproduction in any medium, provided the original work is properly cited.

✔ **Reto M. Baertschiger**

Division of General Surgery, Hospital for Sick Children, University of Toronto, Toronto, ON, Canada

**Summary:**

The article by Fullerton *et al*. describes the results of a pilot study analyzing the outcomes of a cross-regional mentorship program in the field of dementia research over a six-month period. Briefly, the authors demonstrated that early career researchers who participated in a cross-regional (Scotland and London (UCL)) mentorship program found it helpful from a career development and network-building standpoint. Mentors were also satisfied with the program and most importantly both mentees and mentors would participate in a similar program again. Overall, this pilot study demonstrates that mentorship program across institutions or regional barriers is helpful to early career researchers or young investigators in the field of dementia research.

**Comments:**

This pilot study is well-designed and demonstrates that mentorship is crucial for early career researchers. Mentorship can happen across regional, national, and international borders. Mentorship benefits both mentees and mentors, as shown in this study and many others (PMID: 31309744^1^, PMID: 29193588^2^).

As mentioned in their study, one of COVID’s collateral benefits is that most researchers and students are now more comfortable with online communication platforms, facilitating international collaborations and mentorship.

The authors could have described their pairing strategy with more details or a decision tree, to allow the readership to understand how some of the finer pairings were done, especially if the mentees did not suggest any mentors and how they identified fields of expertise of mentors. Did they have any specific liaisons in the subspecialty fields?

The outcome measures seem somewhat short and limited as far as the described "follow-up" is limited to two surveys. Potential career impact, effect on manuscript submission, review, publications, and successful grant applications may be good long-term outcome data to collect.

The introduction and discussion are well-written and documented. Limitations and future directions are excellent as well. The authors should only state their conclusions in that section and limit future research directions and possibilities in their conclusions.

In summary, the manuscript by Fullerton *et al*. describes a pilot study about a new cross-regional mentorship program between Scotland and London and its excellent results with very satisfied mentees and mentors, with real impact on career and personal development, and increased research network for early career researchers in the field of dementia research.

**Is the work clearly and accurately presented and does it cite the current literature?**

Yes

**Is the study design appropriate and is the work technically sound?**

Yes

**Are sufficient details of methods and analysis provided to allow replication by others?**

Yes

**If applicable, is the statistical analysis and its interpretation appropriate?**

Not applicable

**Are all the source data underlying the results available to ensure full reproducibility?**

Yes

**Are the conclusions drawn adequately supported by the results?**

Yes

**Reviewer Expertise:** Basic science cancer research, trauma research, outcomes research in pediatric surgery, chair of an international mentorship program through COG (Children’s Oncology Group)

**I confirm that I have read this submission and believe that I have an appropriate level of expertise to confirm that it is of an acceptable scientific standard.**
